# Drought-Induced Changes in Morphology and Phenology of Olive Trees (*Olea europaea* L.)

**DOI:** 10.3390/plants14233624

**Published:** 2025-11-28

**Authors:** María del Pilar Cordovilla, Yahia Rharrabti, Mohamed El Yamani

**Affiliations:** 1 Center for Advances Studies in Olive Grove and Olive Oils, Faculty of Experimental Science, University of Jaén, Paraje Las Lagunillas, E-23071 Jaén, Spain; mpilar@ujaen.es; 2 Laboratory of Natural Resources and Environment, Polydisciplinary Faculty of Taza, Sidi Mohamed Ben Abdellah University, B.P. 1223, Taza 35000, Morocco; yahia.rharrabti@usmba.ac.ma

**Keywords:** olive tree (*Olea europaea* L.), drought stress, morphology, phenology, climate change, adaptation mechanisms

## Abstract

The olive tree (*Olea europaea* L.), a cornerstone of Mediterranean agriculture, is widely recognized for its inherent drought tolerance. However, the increasing frequency and intensity of water deficit events driven by climate change are challenging its growth, productivity, and long-term sustainability. This review synthesizes current knowledge on the morphological and phenological adaptations of olive trees to water stress. In fact, under drought conditions, olive trees develop a suite of structural and anatomical adjustments that collectively enhance water-use efficiency and help maintain plant water status. These adjustments include reduced leaf area, thickened cuticles, mesophyll rearrangements, remodeling of xylem vessel architecture, and reinforced root systems. These morpho-anatomical responses influence phenology, through changes in the timing and duration of key phenological stages, leading to reduced flower induction, lower flowering intensity, decreased fruit set, and overall lower yields, while the most pronounced effects are observed in sensitive cultivars. Among all stages, flowering is the most vulnerable to water deficit, while pit hardening and fruit development show comparatively more tolerance. The combination of morphological, anatomical, and phenological responses could provide a mechanistic elucidation of drought tolerance variability within olive cultivars. Understanding this interplay is likely to offer valuable criteria in selecting and breeding resistant varieties, thus ensuring productive and sustainable olive cultivation under increasingly severe climatic conditions.

## 1. Introduction

Drought is one of the main environmental factors that determine the plasticity of the morphological, anatomical, physiological, genetic and biochemical traits of plants [1,2,3,4]. Water deficit also significantly influences plant phenological, typically slowing down or altering crucial phases such as budbreak, flowering, and fruit set, with direct implications on yield and overall productivity [5,6].

The Mediterranean region, characterised by long, hot, dry summers and mild, rainy winters, is particularly vulnerable to environmental stress due to the frequency and intensity of drought periods [7,8]. This situation is expected to intensify in the coming decades as a result of climate change, as climate models project an increase in both the frequency and duration and intensity of drought episodes [9].

The olive tree (*Olea europaea* L.) is one of the most representative crops of the Mediterranean basin, largely due to its ability to thrive under water constraints. However, its yield and vegetative development depend largely on the genotype and water availability [10]. In situations of extreme drought, fruit yield, oil quality and production are expected to be seriously compromised [11]. The olive tree’s tolerance to water scarcity is based on a combination of morpho-anatomical, physiological, biochemical and phenological traits, together with mechanisms that regulate water use and enable it to cope with periods of water deficit [6,12,13,14,15,16,17,18]. These adaptations delay the onset of stress and increase tolerance to dehydration [19]. In the ‘Picual’ cultivar, it has been observed that olive trees adapted to arid environments adjust their growth and water absorption capacity by regulating transcription factors, which affects their morphological and physiological responses to drought [20]. Furthermore, the extent of vegetative growth adjustment under water deficit varies depending on the organ affected [21].

Despite extensive research on olive tree responses to water deficit, morphology, anatomy, and phenology are often investigated in isolation. Leaf traits, root characteristics, and phenological stages are frequently analyzed separately, as if they operated independently. This fragmented approach has long obscured the integrated nature of drought adaptation. The present review demonstrates how structural adaptations at the organ level (in leaves, stems, and roots) directly govern the timing and success of critical morphological stages (germination, flowering, and fruit set), thereby shaping overall drought resilience. By viewing these traits as components of a coordinated life-history strategy rather than isolated mechanisms, we provide practical, trait-based criteria for selecting cultivars not merely for survival, but for consistent productivity under water scarcity. This cross-scale synthesis offers a holistic and more predictive perspective than previous fragmented approaches.

This article seeks to summarise current knowledge on the morphological and phenological responses of olive trees to increased water stress associated with climate change. It also analyses the effects of water deficit on morphological traits related to vegetative and reproductive growth and examines alterations in the main phenological phases according to the BBCH (Biologische Bundesanstalt, Bundessortenamt, Chemische Industrie) scale. Finally, it proposes research perspectives aimed at improving the resilience of olive trees to increasingly frequent water stress conditions.

## 2. Effect of Water Stress on Plant Morphology

With regard to morpho-anatomical characteristics, several studies have shown remarkable variability among cultivars in their response to drought [3,22,23]. This is relevant, as olive tree yield depends on factors related to plant architecture, leaf traits and internal microstructural modifications, such as the organisation of dermal, mechanical, vascular and storage tissues [24]. Razouk et al. [25], in a study of 32 cultivars, reported a wide diversity of structural traits associated with water use efficiency. Other authors confirmed that morphological characteristics directly influence yield [21,26]. This variability reflects different levels of plasticity in the olive tree’s response to drought [27]. Therefore, understanding the adaptive mechanisms in different cultivars is essential, especially in climate change scenarios where this crop will be increasingly exposed to water deficits [28].

### 2.1. Leaf Response to Water Stress

According to Edziri et al. [29], the leaf structure of olive trees reflects the effects of drought more clearly than the stem or root. In fact, the leaf is the organ with the greatest capacity to adapt to environmental conditions [30,31]. Under water deficit, morphological and anatomical changes have been documented, such as a reduction in leaf area (LA) and changes in leaf anatomy, which are considered adaptive mechanisms in response to water scarcity [12,17,32].

The vegetative characteristics of olive trees depend on both genotype and water availability [10]. In the ‘Arbequina’, ‘Blanqueta’, ‘Cobrançosa’, ‘Manzanilla’ and ‘Negrinha’ cultivars (Table 1), leaves formed under water stress showed lower LA and specific leaf area (SLA), together with higher leaf density (LD) and leaf mass per unit area (LMA) [12,33]. Similar patterns have been described in the cultivars ‘Chemlali’, ‘Chétoui’, ‘Koroneiki’ and ‘Manzanillo’ [17,34,35] (Table 2). In wild olive trees, water deficit also reduced leaf growth [36]. However, in the ‘Empeltre’ cultivar, no decrease in the number or rate of leaf emergence was recorded [33]. In studies with different cultivars, Amad et al. [26] concluded that a reduction in the number of leaves is associated with a lower transpiration rate, a key factor in yield control [37]. Similarly, Razouk et al. [25], in a study of 32 olive varieties, reported a negative correlation between LA and the leaf stability index against stress, observing a lower reduction in LA in small-leaved cultivars. These findings support the consideration of LA as a good indicator of drought tolerance.

In leaves preformed before the application of water deficit, Marchioni et al. [32] observed a reduction in LA and leaf succulence in the Italian cultivar ‘Degli’ and ‘Leccino’, depending on the severity of the stress, while no changes were detected in ‘Maurino’ (Table 2). In addition, ‘Degli’ showed a decrease in SLA and an increase in LD. The reduction in leaf growth under drought conditions could be related to the decrease in relative water content (RWC) and the limitation in the production of new leaves [38]. These results support the hypothesis that a smaller leaf area contributes to reducing water loss and heat dissipation [32,33,39]. Ahmad et al. [26] noted that leaf size is positively correlated with fruit yield. Furthermore, it has been noted that greater leaf thickness represents a key adaptive trait, as it promotes water conservation in the tissue, improves hydraulic efficiency, optimises reserve function and increases resistance to physical damage from desiccation [40,41,42]. In aged leaves, symptoms of water stress such as chlorosis, loss of turgidity, bronzing, leaf folding and premature leaf fall have been described [38,43].

Leaf anatomy is directly involved in the ability of an olive cultivar to tolerate water stress [12,32]. In a study of various cultivars in semi-arid environments, Ahmad et al. [26] observed that midrib thickness, lamina thickness, phloem thickness, palisade parenchyma cell size, and xylem vessel diameter were closely associated with fruit yield. The proportion of parenchyma tissue in the leaf is a key factor, as it provides more space for water absorption and sugar storage [44]. In this regard, cultivars with a higher percentage of palisade mesophyll are better adapted to drought conditions [12,26,32].

In leaves subjected to water deficit, an increase in the thickness of the palisade parenchyma has been described [12,19,34], even in contact with both epidermis [45]. A palisade parenchyma/spongy parenchyma ratio greater than 1 has been associated with tolerant cultivars, while values less than 1 characterise sensitive cultivars [12,32]. For example, ‘Cobrançosa’ and ‘Maurino’ showed values > 1, consistent with their high tolerance to water deficit (Table 2). This predominance of palisade parenchyma favours photosynthesis even when the stomata close due to drought, increasing CO_2_ assimilation per unit of leaf area [12,19,23,32,46,47,48]. On the other hand, in resistant cultivars such as ‘Chemlali’, an increase in spongy parenchyma has been described, which could facilitate the internal diffusion of CO_2_ [19,49]. In the Tunisian cultivar ‘Meski’, this parenchyma occupied up to three-quarters of the leaf, accompanied by a reduction in palisade parenchyma, an increase in sclereids and greater development of collenchyma, thus reinforcing structural protection [49].

Vascularisation is also a determining factor: greater thickness of the midrib is associated with better transport of photoassimilates and greater storage capacity [50]. In ‘Meski’, thickening of the midrib and increased vascularisation were described, although this was not maintained under severe stress [49]. Under moderate deficit, this cultivar showed remarkable phloem development along with a reduction in woody tissue. In general, a greater number of vascular bundles in the midrib is usually associated with narrower vessels and a lower proportion of sclerenchyma [51,52]. Along these lines, Jacobsen et al. [53] suggested that xylem density can be used as an indicator of drought tolerance in different species.

The size of epidermal cells is also linked to resistance to water deficit. A smaller size in the lower epidermis favours resistance to structural collapse [32,45]. In the ‘Chemlali’ and ‘Chétoui’ cultivars, a reduction in epidermal and mesophyll cell size was recorded, along with an increase in cell density [34,54]. Likewise, in ‘Chemlali’, a thickening of both epidermis was described, associated with greater tolerance compared to ‘Meski’ [19] (Table 2). Added to this is the presence of a thick cuticle and abundant epicuticular wax, characteristics that reduce water loss [12,23,55]. In ‘Arbequina’, ‘Maurino’, ‘Negrinha’ and ‘Meski’, an increase in cuticle thickness was observed under water deficit [12,23,49]. These cuticular layers contribute to the maintenance of turgidity, and several authors have proposed them as an indicator of resistance [25,56]. However, in a study conducted with eleven olive cultivars from different countries and grown in an arid to semi-arid climate, which aimed to relate morpho-anatomical traits to plant yield, a negative correlation was observed between cuticular thickness and fruit yield [26].

Stomatal control is another key factor in water management [57]. Olive trees have small stomata located on the abaxial surface. In the Tunisian cultivars ‘Chemlali’ and ‘Chétoui’, water deficit induced an increase in stomata and trichome density [34]. In ‘Koroneiki’, which is tolerant to water deficit, these increases were greater than in Manzanillo, which is considered sensitive (Table 2). At the same time, the diameter of stomata and trichomes decreased in both varieties under drought conditions [17,58]. Higher stomatal density can promote photosynthetic assimilation by increasing intercellular CO_2_ and allowing better control of transpiration [19,45]. Similarly, an increase in trichome density enhances water use efficiency by increasing boundary layer resistance and reducing transpiration [19,59,60]. This helps to maintain leaf water potential and mitigate growth reduction under drought conditions [17]. On the other hand, Razouk et al. [25] proposed that leaf conductance, measured in detached leaves, can serve as a phenotypic marker of drought tolerance, as it correlates with yield and water use efficiency. According to this criterion, ‘Lechín de Sevilla’, ‘Azeradj’ and ‘Picholine Marocaine’ were classified as tolerant, while ‘Moraiolo’ (Italy), ‘Vernina’ and ‘Frantoio’ were considered sensitive (Table 2).

Together, these morpho-anatomical adaptations help to limit water loss, maintain the water potential of the stem and optimise resource use efficiency. They therefore represent relevant criteria for the selection of cultivars with greater drought resistance [19,32].

### 2.2. Stem Response to Water Stress

In olive trees, the development of vegetative characteristics such as height, canopy size and stem diameter is limited by irrigation restrictions [10,61]. The stem performs essential functions by participating in the transport of water and carbohydrates, as well as acting as a water reservoir [62]. Morphological parameters such as tree height and trunk circumference have been closely associated with productive capacity and fruit yield [21,26].

During drought, reduced water flow from the xylem to adjacent cells causes loss of turgidity, reduced cell elongation, and reduced organ expansion [63]. As a result, olive trees under water stress show a significant decrease in stem height and diameter, with a more severe effect on sensitive cultivars [17,20,25,26,32,35,58,64]. For this reason, the growth rate of stem diameter is considered a reliable indicator of the plant’s water status [58,65]. Recent studies on the ‘Arbequina’, ‘Leccio del Corno’ and ‘Maurino’ cultivars showed that only the latter exhibited reduced stem and shoot growth under water deficit [23] (Table 2). In contrast, Hernández-Santana [21] detected no differences in stem diameter in the ‘Arbequina’ cultivar under similar conditions. Complementarily, McDowell et al. [66] pointed out that, in the absence of water, trees tend to reduce leaf area before trunk thickness as a strategy to limit water consumption. Furthermore, it has been documented that under water deficit conditions, the stem can develop a layer of cork [67,68].

Under drought conditions, the increase in the water potential gradient between the soil and the leaves can limit water transport through the xylem due to cavitation and embolism processes [69]. Susceptibility to these phenomena is closely related to plant resistance. In response to water deficit, an increase in the number of conducting vessels was detected in the ‘Meski’ cultivar, improving vascularity [49]. Similar results were described for the Maurino cultivar [22] (Table 2).

Water dynamics also depend on the anatomical and chemical characteristics of the wood [62,70,71]. Higher wood density has been associated with greater drought tolerance [49,72]. During periods of drought, there may be an increase in lignin deposition in the xylem vessels [71], which reduces susceptibility to embolism [62]. Likewise, a higher S/G ratio of lignin confers additional resistance, given that S-rich lignin has more flexible and hydrophilic properties [62].

In olive trees, the presence of small-diameter vessels increases drought resistance by protecting against cavitation and preventing vessel collapse [73,74,75]. Rossi et al. [76] observed in the Italian cultivar ‘Nocellara del Belice’ (Table 1) that, as an immediate response to low water availability, the diameter of xylem vessels was reduced, constituting an adaptive mechanism. In the ‘Meski’ cultivar, water stress reduced the proportion of wood and increased the development of parenchyma and cortical collenchyma, accompanied by an increase in sclerenchyma fibres and the phloematic zone [49]. A positive correlation between fruit yield and the thickness of the collenchyma and phloem has also been documented [26]. The increase in sclerenchyma tissues around the vascular bundle helps to prevent the collapse of the conducting elements under turgor loss [77]. In some cultivars, increased sclerenchyma thickness under drought conditions has also been reported, along with a reduction in the phloem zone [26]. In addition, the ‘Meski’ cultivar accumulated abundant starch grains in the stem pith, which is interpreted as an adaptive mechanism to improve stress tolerance through the mobilisation of reserves [49,78].

### 2.3. Root Response to Water Stress

The roots of plants subjected to drought can develop morphological, anatomical and physiological adaptations that favour their survival [79]. Under rainfed conditions, olive trees adjust the root/leaf ratio to explore a greater volume of soil and optimise water and nutrient uptake [36,80]. For this reason, rainfed olive trees tend to have higher root/crown ratios than irrigated ones [81,82,83]. Under water deficit conditions, an increase in root density and root/stem ratio has been described [34,84], in contrast to what has been reported in other woody species [85]. Likewise, in olive trees, a reduction in the ratio between total root length and root dry mass has been observed, a characteristic linked to the ability to penetrate more compact soil horizons [86]. In wild olive trees, water deficit reduced root growth and decreased the leaf area/sapwood area and leaf area/root area ratios [36]. Under these conditions, the development of longer roots facilitates water absorption in deep layers, which allows significant transpiration flow to be maintained and constitutes a key survival mechanism [19,58,87]. In the Arbequina cultivar, root architecture showed adaptations to stress by exploring larger volumes of soil and shifting towards the formation of finer roots within the moist bulb [88].

Several studies have shown that the radial hydraulic conductivity of the root decreases under drought conditions [89,90,91,92]. The transfer of water from the soil to the xylem is regulated by the presence of apoplastic barriers, whose formation is associated with suberisation processes. Thus, reduced transpiration and the formation of these barriers limit apoplastic flow, favouring the symplastic pathway, in which aquaporins play a central role [20,79,93].

Suberin accumulation is inversely related to root hydraulic conductivity. In the Italian cultivars ‘Biancolilla’ (sensitive) and ‘Coratina’ (tolerant), a marked increase in suberisation was observed in the exodermis and endodermis, where the number of suberised cell layers increased between 3.5 and 6 times under drought conditions [94] (Table 2). Similar results were described in the ‘Meski’ cultivar [49]. In addition, suberisation reduces water loss through passive flow into the soil, preserving the vitality of root meristems and promoting the formation of secondary roots [94,95].

**Table 2:** Relative tolerance of cultivars and main morpho-anatomical characteristics studied.

Relative Cultivar Tolerance	Morpho-Anatomical Traits Studied	Reference
Manzanilla, Negrinha, Cobrançosa > Arbequina, Blanqueta	Leaf area, leaf tissue thickness, stomatal density, density of leaf tissue (high palisade/spongy parenchyma ratio), cuticle thickness, epidermis thickness, and trichome layer thickness	[3]
Chemlali > Chétoui	Leaf area, density, density of leaf tissue, stomatal density, trichome density, thickness of epidermis, and cuticle	[34]
Chemlali > Meski	Leaf area, leaf tissue thickness, stomatal density, density of leaf tissue, epidermis thickness, stomatal density, trichome density	[19]
Coratina > Biancolilla	Root morpho-anatomical parameters: cell wall suberization degree, root section circularity index, intercellular spaces area, cell number per unit area, cell size	[94]
Koroneiki > Manzanillo	Leaf area, stomatal density, and dimensions (length and width) and trichome density and diameter, trunk cross-sectional area, plant height	[17]
Empeltre > Arbequina	Leaf area, leaf appearance rate, total number of leaves per plant, growth after water stress	[33]
Dezful, Konservolia > Zard Aliabad, Roughani, Shengeh, Manzanilla, Sevillana, Mission	Shoot and fruit length and diameter	[61]
Lechín de Sevilla, Azeradj, Picholine Marocaine > Leccino > Chetoui > Arbequina, Blanqueta, Maurino > Madural > Sevillenca, Coratina > Moraiolo, Vernina, Frantoio	Leaf area, leafing intensity, fruit weight, petiole elasticity, stomatal density, stomatal length, trichomes density, trichome width, trichomes per stoma, trichome area index	[25]
Erlik > Hamdi > BARI-2, HP Olive, QR Olive, FS-17, Nabali, Gemlik, Souri, Manzanilla	Plant traits evaluated included plant height, trunk circumference, number of leaves and fruits, and fruit length, diameter, and weight. Root: thickness of the thinnest cortical region, cortical cell area, collenchymatous and sclerenchymatous layers, phloem and epidermal thickness, metaxylem area, and total cross-sectional area. Stem: cross-sectional area, epidermal and cortical region thickness, collenchymatous and sclerenchymatous layer, phloem thickness, cortical cell area, and metaxylem vessel diameter. Leaf: length, width and area, lamina, spongy and palisade parenchyma thickness, cuticle and epidermal thickness, and metaxylem vessel and phloem thickness	[26]
Maurino > Leccino > Degli	Leaf area, epicuticular waxes thickness, cuticle thickness, longitudinal diameter of epidermis cells, density of leaf tissue, shoot and fruit length	[32]
Leccio Corno > Arbequina > Maurino	Leaf area, leaf tissue thickness, cuticle thickness, density of leaf tissue, epidermis thickness, shoot length, stem diameter, vulnerability to xylem embolism formation	[23]

The reduction in root conductivity may also be associated with morphological changes, such as reduced root circularity due to deformation caused by dehydration and solute accumulation [80,94,96]. In olive trees, drought causes the cessation of mitotic activity in cortical cells, an increase in intercellular spaces due to cell collapse, and greater development of the pith [49,94]. It should be noted that greater cortical thickness has been associated with greater absorption capacity [26]. Likewise, the proportion of parenchyma cells in the pith and cortex is linked to greater stress tolerance, as it facilitates the storage and transport of solutes [97]. Ahmad et al. [26] reported a negative correlation between fruit yield and variables such as cortical thickness, cortical cell area, collenchyma thickness, and medullary cell area. In contrast, they observed a positive correlation between root phloem thickness and fruit yield.

In the ‘Meski’ cultivar, under moderate water stress, an increase in sclerenchymatic fibres was recorded, invading the cortex and forming a continuous ring around the phloem. Under severe drought, this phenomenon intensified along with an increase in the number of woody vessels, contributing to root thickening. Starch accumulation was also detected in the cortex [49]. This sclerification process is linked to greater mechanical reinforcement of the root and greater penetration capacity in compact soils [98,99].

Finally, Ahmad et al. [26] reported an increase in epidermal thickness in the cultivars ‘BARI-2’, ‘HP Olive’, ‘QR Olive’, ‘Erlik’, ‘Hamdi’, ‘FS-17’, ‘Nabali’, ‘Gemlik’, ‘Souri’ and ‘Manzanilla’ under water deficit (Table 2).

### 2.4. Response of Flowering and Fruit Formation to Water Stress

Water deficit during flowering is a determining factor in final production, as it reduces both the number of fruits and the oil content [100]. In the ‘Arbequina’ cultivar, deficit irrigation in spring significantly reduced the number of inflorescences per shoot, fertile inflorescences and fruits per shoot, as well as total yield [100,101]. Similar results were obtained in the ‘Koroneiki’ (Greece) and ‘Morisca’ (Spain) cultivars [102,103]. This reduction can be attributed to the intensification of natural flower and fruit drop [100]. Fruit drop increases when there is water deficit from flowering to endocarp hardening [100]. Likewise, fruit oil content has been related to the dry weight reached when the stone hardens and to the water potential of the stem during flowering [100]. In contrast, in the Arbosana cultivar, Trentacoste et al. [104] reported an increase in productivity under spring water deficit. These differences could be explained by the asymptotic relationships between oil production, water applied, and the water status of the plant [64,101].

The reduction in vegetative growth during sensitive phases under drought conditions also decreases fruit production and yield [105]. In studies conducted by Razouk et al. [25] on several cultivars (‘Picholine Marocaine’, ‘Arbequina’, ‘Frantoio’, ‘Madural’ and ‘Sevillenca’; Table 1), water stress reduced yield, with this effect being less pronounced in the more tolerant cultivars (such as ‘Picholine Marocaine’) (Table 2). This decrease was related to fruit weight loss and premature fruit drop. Similar results were reported in Frantoio and Arbequina [106,107], as well as in Manzanilla de Sevilla [108].

On the other hand, Mezghani et al. [109] reported that in some varieties (‘Coratina’, ‘Manzanilla’ and ‘Chemlali’) fruit weight did not change under water stress, while in others it even increased (‘Picholine’ and ‘Chetoui’). In contrast, Gholami et al. [61] observed that in the cultivars, drought reduced fruit weight, length and diameter. The same study recorded an increase in dry matter under irrigation deficit, probably associated with a reduction in relative water content. In general, the negative impact of drought on fruit growth is more pronounced in sensitive cultivars [11].

## 3. Linking Morphological and Anatomical Adjustments to Phenological Responses of Olive Trees Under Drought Stress

The morphological and anatomical adjustments described above form the functional basis for understanding how water deficit shapes olive tree phenology. Drought-induced modifications in leaf structure, xylem anatomy, hydraulic conductance, and carbon storage directly influence the timing and success of key reproductive events such as budbreak, flowering, and fruit set. Field and controlled experiments consistently show that spring water deficits reduce floral differentiation, increase flower and fruit drop, and limit shoot elongation, whereas controlled deficits during later phenological stages tend to have milder effects on final yield [5,14,17,32].

Spring water stress reduces vegetative growth prior to floral induction, thereby decreasing the number of floral buds and the overall intensity of flowering [5,17]. During fruit set, it affects pollination, initial fruit expansion, and final fruit size. In the ‘Arbequina’ cultivar, spring irrigation restrictions significantly reduced flowering, fruit set, and yield [5]. These changes reflect the interaction among hydraulic regulation, assimilate availability, and carbon reserve levels, linking functional anatomy with reproductive phenology. Hydraulic transport plays a central role in this interaction. In fact, reduced root conductance and xylem anatomical changes limit water supply to growing shoots, inflorescences, and young fruits, thereby compromising turgor and cell expansion necessary for reproduction [14,32]. Cultivars with larger vessels, although more conductive, are more vulnerable to embolism during drought, which restricts early growth and fruit set success [14]. Maintaining starch reserves in leaves and storage tissues helps sustain flowering and fruit set when photosynthesis declines under water stress, with marked genotypic differences [32]. Furthermore, effective stomatal regulation and higher photosynthetic efficiency enable some genotypes to maintain reproductive performance despite moderate water deficits [13].

Allometric adjustments, particularly the reduction in leaf area relative to sapwood or root biomass, reduce transpiration requirements and help preserve turgor in reproductive organs during drought, thereby mitigating negative effects on flowering and fruit set [13].

**Figure 1:**
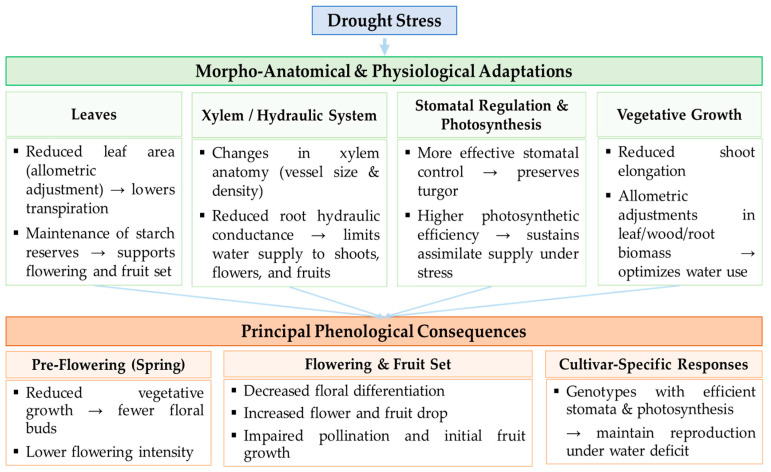
Linking olive tree drought stress adaptations to phenological outcomes.

Together, these integrated morphological, anatomical, and physiological responses explain cultivar-specific sensitivity to water stress and provide the mechanistic link between structural adaptation and phenological performance (Figure 1).

## 4. Phenological Adaptations of Olive Trees to Drought Stress and Agronomic Implications

Olive-growing is of major economic relevance, especially in the Mediterranean region. It is strongly affected by environmental conditions such as temperature, rainfall and water availability [110]. Current climate variations, linked to global change, are starting to impact agro-ecosystems in many areas of the planet [111]. The Mediterranean basin, a particularly vulnerable zone, is facing a rise in temperatures, a drop in rainfall and an intensification of meteorological events, with prolonged periods of drought and occurrences of extreme rain [112]. These changes are likely to significantly compromise both the quality and quantity of olive yields [113,114,115], by affecting essential physiological processes [116], together with its phenological calendar [117,118]. A rigorous understanding of the olive tree phenological cycle and its development phases, in relation to environmental variations, is integral to optimal orchard management, notably in matters of irrigation, fertilization and pest control [119]. This also serves as a strategic instrument for addressing the issues raised by climate change.

### 4.1. Key Phenological Stages of the Olive Tree

The phenological growth stages of olive trees are generally described using three major systems [120,121,122], all conceived in specific contexts and for specific purposes.

Colbrant & Fabres [120] were among the first to propose a detailed phenological scale for olive trees, giving a precise description of the annual cycle divided into several vegetative and reproductive stages. They identified the subsequent key phases: winter dormancy (vegetative rest of terminal and axillary buds), vegetative budbreak (bud swelling), formation of floral clusters (appearance of floral buds), swelling of floral buds (buds round out and swell), corolla swelling and differentiation (separation of calyx and corolla), beginning and full flowering with petal fall, fruit set (formation and early growth of fruit), and finally ripening (veraison and ripening of fruit). This oldest system is still widely used in the Mediterranean region, due to its practicality and ease of application in the field.

The phenological scale developed by De Andrés [121] is a system that has been proposed to describe and monitor the development stages of fruit trees, along with olive trees, based on observable morphological changes. Initially focused on stages related to inflorescence formation and flowering, it did not provide a detailed account of the whole tree development cycle. Indeed, De Andrés system consists of 13 phenological stages, mainly relating to inflorescence formation and flowering, with a less precise description of fruit development, as well as bud and leaf growth stages. Though less comprehensive and standardized, the De Andrés scale is still a valuable historical reference, particularly in Spanish-speaking regions, where its descriptive approach makes it suitable for studying the flowering of olive trees and assessing the impact of climatic conditions on vegetative cycles. It has also served as a useful basis for modern phenological methods and agronomic research. However, due to his limitations, more detailed and widely applicable systems have been developed, notably the BBCH scale, which is now regarded as the standard. The BBCH scale was adapted to olive trees by Sanz-Cortés et al. [122] in order to standardize the description of their phenological stages, based on the code initially developed for cereals by Zadoks et al. [123]. The BBCH code, structured as a two-digit system, describes eight main stages of olive tree development covering bud, leaf, and shoot growth, inflorescence emergence, flowering, fruit development, fruit maturity, and senescence as well as 32 secondary sub-stages. The first digit (0 to 9) corresponds to the main stage, while the second digit (0 to 9) specifies the sub-stage within each phase, allowing for a detailed and systematic characterization of the phenological cycle (Table 3).

### 4.2. Impact of Drought Stress on Olive Tree Phenology

The olive tree has a set of adaptation mechanisms that give it considerable resilience to drought stress [56,67]. Nevertheless, recent investigations emphasize that intensifying water deficits, arising from climate change through reduced spring rainfall and increased temperatures, cause significant physiological, biochemical, and morphological disorders [116,124]. These disturbances directly affect its phenological development, with the magnitude of the impact greatly varying across phenological stage and among cultivars that exhibit distinct levels of sensitivity to water deficit [6,117,125].

Winter dormancy is a key phase of physiological rest in the annual phenological cycle of the olive tree, during which metabolic activity is significantly reduced, allowing carbohydrate reserves to be reconstituted and buds to be prepared for spring budbreak prior to vegetative growth [67,117,124]. This period begins with deep dormancy (BBCH 00–09), followed by early bud reactivation, including shoot elongation (10–19), leaf development (20–29), lateral shoot formation (30–39), and flower bud differentiation and inflorescence development (40–49), terminating at the pre-flowering stage (BBCH 50) [117].

Budbreak (BBCH 51) marks the end of this dormancy and the active resumption of vegetative growth. This crucial phenological stage governs both floral differentiation and the development of fertile shoots. However, budbreak has been found to be particularly sensitive to water availability in late winter and early spring, a period when water deficit can disturb dormancy release, delay initial growth, and consequently impair both floral and vegetative development of olive trees [126,127].

Numerous studies have documented that water stress during this stage significantly retards phenological progression. García-Mozo et al. [117] conducted a 17-year study (1996–2012) at ten sites in Córdoba province (Spain). They observed a similar phenological pattern across all sites, but with a 24-day delay in the emergence of inflorescences (budbreak), coinciding with a 1.5 °C increase in spring temperature, 11 fewer rainy days in spring, and a 150 mm decrease in total precipitation. The authors also noted that the regional delay in budbreak does not affect the two local cultivars ‘Hojiblanca’ and ‘Picudo’ in the same way. ‘Hojiblanca’ delays dormancy release, under water deficit, adopting a conservative strategy suited dry climates, whereas ‘Picudo’ breaks bud earlier and responds more sensitively to thermal fluctuations, making it more vulnerable to early-season drought. Brito et al. [116] reported that late winter water stress slows olive budbreak by reducing cell turgidity and bud growth. This delay likely results from multiple interconnected mechanisms. Water stress limits the cellular rehydration of floral buds and interferes the accumulation of chilling units required for complete dormancy release [116]. Benlloch-González et al. [113] attribute this retardation to decreased cell turgor and hormonal change, specifically an increase in abscisic acid levels coupled with reduced cytokinin synthesis, which collectively inhibit the differentiation of floral buds. Accordingly, vegetative growth is also impaired, leading to a significant loss of shoot length and leaf area [128]. Such phenological delays cause a desynchronization between vegetative development and optimal climatic conditions, potentially shortening the growing season and compromising subsequent phenological stages [6]. Recently, Didevarasl et al. [18] explored the potential impact of climate change on the phenological stages of olive trees in the Euro-Mediterranean region. Unlike previous findings, they project that budbreak will occur earlier under future warming, advancing by an average of 8 to 13 days by 2050. Unexpectedly, mid-late cultivars (e.g., Frantoio, Moraiolo) are expected to exhibit a slightly greater shift than early cultivars (e.g., Carolea, Picholinare), particularly under the RCP 8.5 scenario. This advance is expected to vary across regions, being more pronounced in colder areas such as Northern Europe, where warming will promote rapid emergence from dormancy.

As for flowering (BBCH 60–69), it is widely considered one of the most sensitive phenological phases to water stress in olive trees, particularly during the spring period (April–May) [113,124]. Indeed, Rapoport et al. [5] conducted an investigation on young ‘Picual’ olive trees, revealing that controlled water deficit (irrigation reduced to 25% of evapotranspiration) strongly affected flowering. During inflorescence development, water deficit reduced the number of inflorescences, total and perfect flowers, and impaired ovule development. When applied just before flowering, it limited starch reserves in the ovary and ovule, thereby weakening the flowers. Finally, during flowering, many flowers remained closed, preventing fertilization and reducing fruit production. Similarly, during flowering, water stress (Ψstem < –1.5 MPa) reduced the number of inflorescences per bud, inflorescence fertility, and the number of fruits per bud in the Arbequina cultivar [100]. Didevarasl et al. [6] noted that a 30–50% reduction in effective flowering can occur under moderate to severe water stress, resulting in a significant drop in production potential. Garcia-Mozo et al. [117] observed an average advancement of 1.7 to 1.9 days per year in BBCH 61–65 between 1996 and 2012, combined with a shortening of total flowering duration by nearly 38 days. The same authors reported contrasting responses to water deficit between the two studied cultivars. ‘Picudo’, which is better adapted to cold and dry areas at high-altitude, exhibited earlier flowering that was more sensitive to fluctuations in water and temperature. In contrast, ‘Hojiblanca’, prevalent at lower altitudes, showed more stable flowering under water stress, reflecting a higher tolerance to warm and dry conditions. This phenological desynchronization is associated with lower spring precipitation and higher minimum temperatures, which alter optimal pollination conditions [129,130]. These phenological observations reflect several physiological changes. In fact, spring water stress induces stomatal closure, which in turn reduces photosynthesis and restricts the availability of assimilates needed for forming and maintaining flowers [67,124]. Consequently, this leads to a significant decline in the number of inflorescences and flowers [124], and in the proportion of viable hermaphroditic flowers [113], as well as an alteration in pollen viability and pollen tube germination [113].

Flowering is followed by fruit set (BBCH 69–75), a critical phenological phase marking the transition from fertilized flowers to developing young fruits. This process depends not only on successful fertilization, but also on the ability of the tree to prevent early fruit abscission, a phenomenon closely influenced by the plant’s water status [124]. Under spring water deficit, the later-flowering ‘Hojiblanca’ is more exposed to late-spring stress that reduces fruit set, while the earlier-setting ‘Picudo’ can partly avoid it if April rainfall is sufficient, although May–June drying remains a major factor limiting yield [117]. Severe water stress significantly reduced fruit set in the ‘Picual’ cultivar, initially by limiting starch reserves needed for fertilization, and subsequently by preventing flower opening, leaving flowers closed and unpollinated, ultimately causing a drastic drop in fruit set [5]. Benlloch-González et al. [113] have noticed that under moderate to severe water stress, olive trees can lose 40 to 60% of their fruit set. This disturbance manifests as a massive drop in young fruit, known as ‘green drop’, which constitutes a self-regulatory mechanism allowing the tree to allocate its resources on a limited number of viable fruits [130]. Physiologically, this drop is linked to a reduced cell turgor and a hormonal imbalance affecting abscission tissues [128]. Furthermore, the impact of water stress on fruit set is considerably exacerbated when accompanied by heat stress, a situation that is increasingly frequent in Mediterranean olive groves, thereby worsening fruit loss and putting the final yield at greater disadvantage [130]. Meanwhile, Siakou et al. [131] reported that the application of water deficit (irrigation at 35% of evapotranspiration) had no effect on fruit set in the ‘Koroneiki’ cultivar.

As for pit hardening and fruit development (BBCH stages 75–77), which generally occur in full summer (July-August), the literature states that they are relatively tolerant to water stress, albeit able to be affected by severe stress, in contrast to the earlier phenological stages that are much more sensitive [132]. Indeed, it has been claimed that at these stages, olive trees can support moderate or controlled water deficits without adverse effects on final yield, and may even tolerate a leaf water potential of −4.0 MPa without irreversible damage [106,128,133,134]. This physiological resistance justifies the use of regulated deficit irrigation strategies that help to reduce water use by 25–50% [131]. However, this tolerance has its limits, and other authors have recorded that under severe summer water stress, the duration of pit hardening can be reduced by 25 to 30%, causing a significant reduction in fruit size and weight [6,116]. This reduction results from an inhibition of cell division in the mesocarp and a hormonal imbalance between abscisic acid and auxins, which interfere with fruit tissue growth [132,135]. In line with these findings, Garcia-Mozo et al. [117] noted that pit hardening is sensitive to water stress, which can affect fruit growth and the final size of olives. During the period from flowering to pit hardening, water stress (Ψstem < –1.8 MPa) further reduces fruit dry mass and, consequently, fruit size, and it also decreases oil content in the ‘Arbequina’ cultivar [100].

Finally, with regard to maturity of the fruit (BBCH 80–89), a defining phase for lipid accumulation and phenolic compound synthesis, water stress exerts contrasting effects as a function of its intensity and duration. Actually, a moderate deficit can improve the concentration of phenolic compounds, such as oleuropein and verbascoside, thus enhancing the oxidative stability and antioxidant properties of the oil, especially when stress is applied prior to pit lignification [136,137]. On the other hand, severe or prolonged stress accelerates leaf senescence, reduces fruit filling period, and restricts lipid biosynthesis, driving down olive oil yield by 20 to 50% and decreasing the proportion of unsaturated fatty acids [67,138]. These disorders are matched by a decline in photosynthesis activity and assimilate translocation to the fruit [139]. In addition, several authors have reported that the shortening of vegetative cycles is worsened by phenological shifts related to global warming, exposing fruits to more intense summer droughts and thus increasing interannual yield variability and olive grove vulnerability [6,130,140]. Therefore, although water stress diminishes the amount of oil extracted, it also enhances certain qualitative aspects, highlighting the need for precise irrigation management to balance yield and quality [137,139]. Fruit maturity in both ‘Picudo’ and ‘Hojiblanca’ is influenced by reduced precipitation and rising temperatures, conditions that accelerate ripening but compromise fruit quality and size, potentially lowering final yield [117]. Consistent with this decline in performance under water stress, a 66% reduction in oil production was reported in ‘Arbequina’ for a decrease of just 1 MPa in midday stem water potential [100].

## 5. Conclusions

This review shows that, under conditions of water deficit, olive trees develop a wide range of morphological and anatomical responses aimed at improving water use efficiency and maintaining their water potential. In the leaves, these responses include reduced leaf area, increased epidermal cell density, thickening of the cuticle, higher stomatal and trichome density, along with modifications in the mesophyll that help sustain photosynthetic activity during drought. Stem growth, both in height and diameter, decreases under water stress, particularly in sensitive cultivars. Greater tolerance is related to the development of more numerous xylem vessels with smaller diameters, increased lignin deposition, a higher S/G ratio, and an increased in sclerenchymatic tissues, all of which contribute to reducing cavitation and preventing the collapse of conducting vessels. In the roots, water stress promotes increased root density as well as the formation of longer and thinner roots, thereby optimizing water uptake. Sclerification increases mechanical resistance and facilitates penetration into compacted soils, while suberisation reduces passive water loss, preserves the vitality of root meristems, and promotes the formation of secondary roots. Taken together, the morphological and anatomical adaptations described across plant organs represent fundamental criteria for the selection and improvement of cultivars with greater tolerance to water stress.

In terms of phenology, drought frequently delays or shortens key developmental stages such as budbreak, flowering, and fruit set, redirecting resources toward maintenance at the expense of reproductive success. As a result, water deficit during both vegetative growth and flowering significantly reduces flower number, fruit set, and final yield, ultimately compromising both oil quantity and quality. These effects are generally more pronounced in drought-sensitive cultivars, underscoring the need to identify and promote genetic materials with greater resilience under water-limited conditions. At the same time, these phenological shifts illustrate the capacity of olive trees to balance survival and productivity, an adaptive resilience that will be increasingly tested as climate change intensifies drought frequency and severity.

Looking ahead, holistic approaches involving morphology, anatomy, ecophysiology, biochemistry, and phenology will be required to fully understand olive tree responses to water stress and anticipate its impacts. Coupling these disciplines with genomics and remote sensing will help improve phenological behavior modeling and identify critical water stress thresholds. Meanwhile, combining sustainable irrigation strategies with biotechnological innovations can enhance water use efficiency and strengthen the long-term resilience of olive groves in increasingly dry climatic conditions.

## Figures and Tables

**Table 1:** Olive varieties mentioned in this review and their geographical origins.

Cultivar	Origin	Cultivar	Origin
Arbequina	Spain	Lechín de Sevilla	Spain
Azeradj	Algeria	Madural	Portugal
BARI-2	Pakistan	Manzanilla	Spain
Biancolilla	Italy	Manzanillo	Spain
Blanqueta	Spain	Maurino	Italy
Chemlali	Tunisia	Meski	Tunisia
Chétoui	Tunisia	Mission	United States
Cobrançosa	Portugal	Moraiolo	Italy
Coratina	Italy	Morisca	Spain
Degli	Italy	Nabali	Palestine
Dezful	Iran	Negrinha	Portugal
Empeltre	Spain	Nocellara del Belice	Italy
Erlik	Israel	Picholine Marocaine	Morocco
Frantoio	Italy	QR Olive	Pakistan
FS-17	Italy	Roughani	Iran
Gemlik	Turkey	Sevillana	Spain
Hamdi	Tunisia	Sevillenca	Spain
HP Olive	Pakistan	Shengeh	Iran
Konservolia	Greece	Souri	Lebanon
Koroneiki	Greece	Vernina	Italy
Leccino	Italy	Zard Aliabad	Iran
Leccio del Corno	Italy		

**Table 3:** Phenological stages of the olive tree based on the BBCH scale, adapted from Sanz-Cortés et al. [122].

BBCH Code		Description
**Principal Stage 0:**		**Bud development**
*Secondary Stages* *(Sec. Stgs)*	00	Foliar buds at the apex of shoots grown the previous crop-year are completely closed, sharp-pointed, stemless and ochre-coloured.
	01	Foliar buds start to swell and open, showing the new foliar primordia.
	03	Foliar buds lengthen and separate from the base.
	07	External small leaves open, not totally separated, remaining joined at apices.
	09	External small leaves opening further with their tips inter-crossing.
**Principal Stage 1:**		**Leaf development**
*Sec. Stgs*	11	First leaves are fully separated and exhibit a greenish-grey colour
	15	Leaves are longer without attaining final length. First leaves turn greenish on the upper side.
	19	Leaves achieve typical cultivar length and shape.
	31	Shoots reach 10% of final length.
**Principal Stage 3:**		**Shoot development**
*Sec. Stgs*	33	Shoots achieve 30% of final length.
	37	Shoots achieve 70% of final length.
**Principal Stage 5:**		**Inflorescence emergence**
*Sec. Stgs*	50	Flower buds in the leaf axils are fully closed, sharp-pointed, stemless, and ochre- coloured.
	51	Inflorescence buds begin to swell.
	52	Inflorescence buds open and development of flower clusters begins.
	54	Flower cluster growing.
	55	Flower cluster completely expanded. Floral buds start to open.
	57	Corolla green-coloured, longer than calyx.
	59	Corolla changes from green to white.
**Principal Stage 6:**		**Flowering**
*Sec. Stgs*	60	First flowers open.
	61	Start of flowering: 10% of flowers open.
	65	Full flowering: at least 50% of flowers open.
	67	First petals falling.
	68	Majority of petals fallen or faded.
	69	End of flowering, fruit set, unfertilised ovaries fallen.
**Principal Stage 7:**		**Fruit development**
*Sec. Stgs*	71	Fruit reaches ~10% of its final size.
	75	Fruit reaches ~50% of its final size. Stone (endocarp) becomes lignified (resistant to cutting).
	79	Fruit ~90% of final size and is suitable for green picking.
**Principal Stage 8:**		**Maturity of fruit**
*Sec. Stgs*	80	Fruit colour changes from deep green to light green or yellowish.
	81	Fruit begins to color
	85	Specific fruit coloring increases.
	89	Harvest maturity: fruits achieve the typical cultivar colour, remain turgid and are suitable for oil extraction.
**Principal Stage 9:**		**Senescence**
*Sec. Stg*	92	Overripe: Fruits lose turgidity and begin to drop.

## Data Availability

No new data were created or analyzed in this study.
